# Ultrathin All‐Solid‐State MoS_2_‐Based Electrolyte Gated Synaptic Transistor with Tunable Organic–Inorganic Hybrid Film

**DOI:** 10.1002/advs.202308847

**Published:** 2024-04-02

**Authors:** Jungyeop Oh, Seohak Park, Sang Hun Lee, Sungkyu Kim, Hyeonji Lee, Changhyeon Lee, Woonggi Hong, Jun‐Hwe Cha, Mingu Kang, Jun Hyup Jin, Sung Gap Im, Min Ju Kim, Sung‐Yool Choi

**Affiliations:** ^1^ School of Electrical Engineering Graduate School of Semiconductor Technology Korea Advanced Institute of Science and Technology (KAIST) 291 Daehak‐ro, Yuseong‐gu Daejeon 34141 Republic of Korea; ^2^ Department of Nanotechnology and Advanced Materials Engineering Sejong University 209 Neungdong‐ro, Gwangjin‐gu Seoul 05006 Republic of Korea; ^3^ Department of Chemical and Biomolecular Engineering Graphene/2D Materials Research Center Korea Advanced Institute of Science and Technology (KAIST) 291 Daehak‐ro, Yuseong‐gu Daejeon 34141 Republic of Korea; ^4^ School of Electronics and Electrical Engineering Dankook University Gyeonggi 16890 Republic of Korea

**Keywords:** artificial synapse, deep neural network, electrolyte gated transistor, initiated chemical vapor deposition, molybdenum disulfide

## Abstract

Electrolyte‐gated synaptic transistors (EGSTs) have attracted considerable attention as synaptic devices owing to their adjustable conductance, low power consumption, and multi‐state storage capabilities. To demonstrate high‐density EGST arrays, 2D materials are recommended owing to their excellent electrical properties and ultrathin profile. However, widespread implementation of 2D‐based EGSTs has challenges in achieving large‐area channel growth and finding compatible nanoscale solid electrolytes. This study demonstrates large‐scale process‐compatible, all‐solid‐state EGSTs utilizing molybdenum disulfide (MoS_2_) channels grown through chemical vapor deposition (CVD) and sub‐30 nm organic‐inorganic hybrid electrolyte polymers synthesized via initiated chemical vapor deposition (iCVD). The iCVD technique enables precise modulation of the hydroxyl group density in the hybrid matrix, allowing the modulation of proton conduction, resulting in adjustable synaptic performance. By leveraging the tunable iCVD‐based hybrid electrolyte, the fabricated EGSTs achieve remarkable attributes: a wide on/off ratio of 10^9^, state retention exceeding 10^3^, and linear conductance updates. Additionally, the device exhibits endurance surpassing 5 × 10^4^ cycles, while maintaining a low energy consumption of 200 fJ/spike. To evaluate the practicality of these EGSTs, a subset of devices is employed in system‐level simulations of MNIST handwritten digit recognition, yielding a recognition rate of 93.2%.

## Graphical abstract

This study explores ultrathin all solid‐state electrolyte‐gated synaptic transistors (EGSTs) with chemical vapor deposition (CVD) grown molybdenum disulfide (MoS_2_) channel and initiated chemical vapor deposition (iCVD) grown organic–inorganic hybrid electrolyte. The fabricated MoS_2_‐based EGST achieves outstanding synaptics properties, benefitting from sub‐30 nm ultrathin electrolyte and atomically thin channel. The approach provides a guide for EGSTs to overcome scaling challenge.

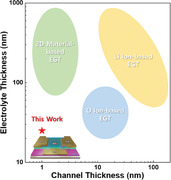

## 1 Introduction

With the emergence of the big data and continuous advancements in computer performance driven by Moore's law, artificial intelligence (AI) has achieved remarkable progress not only in tasks such as recognition and classification^[^ ^1^, ^2^, ^3^ ^]^ but also in creative endeavors such as DALL‐E2 for image generation and ChatGPT for conversational AI.^[^ ^4^, ^5^, ^6^ ^]^ However, the implementation of high‐level intelligent tasks requires neural networks with complex structures composed of a large number of neurons and synapses, which leads to an exponential increase in the number of network parameters.^[^ ^7^, ^8^ ^]^ Unfortunately, the traditional von Neumann architecture, which stores and processes data in separate physical regions, suffers from the time‐ and energy‐consuming task of transferring a vast number of network parameters.^[^ ^9^, ^10^ ^]^ To address this challenge, neuromorphic computing has attracted considerable attention. This enables data storage and processing in the memory space. Neuromorphic computing leverages synaptic devices located at the intersections of crossbar arrays to enable parallel multiply and‐accumulate (MAC) operations, which are the most computationally demanding tasks in deep neural networks.^[^ ^7^ ^]^ Synaptic devices that primarily store analog resistance have been demonstrated using 2‐terminal memory devices, such as phase‐change memory,^[^ ^11^, ^12^ ^]^ magnetic random access memory,^[^ ^13^ ^]^ and resistive random access memory,^[^ ^14^, ^15^ ^]^ However, they encounter issues such as the sneak path problem and read/write coupling, leading to instability in their states.^[^ ^16^ ^]^ As a solution, three‐terminal synaptic devices incorporating conductance programming gates have been actively investigated. Examples include floating‐gate memory,^[^ ^17^ ^]^ charge‐trap memory,^[^ ^18^ ^]^ ferroelectric field‐effect transistors,^[^ ^19^ ^]^ and electrolyte‐gated transistors (EGT).^[^ ^20^ ^]^ These devices perform their read and write functions through physically separate terminals, thereby alleviating the aforementioned issues.

One of the most promising candidates for synaptic devices is the electrolyte‐gated synaptic transistor (EGST), which stands out because of its simple device structure and remarkable synaptic performance, including linear weight updates, low power operation, and excellent endurance.^[^ ^21^, ^22^ ^]^ EGST utilizes electrically insulating electrolytes, which serve as an ionic conductor, to modulate the distribution of a cation (Li^+^ of H^+^) or anion (O^−^) toward or away from the channel. Among them, H^+^‐based EGST has great potential to demonstrate the ideal synaptic device by its great CMOS compatibility and fast proton migration. The proton containing electrolyte leads to the formation of an extremely thin electrical double layer (EDL) with a thickness of ≈1 nm at the interface between the electrolyte and channel. The ultrathin EDL generates superior capacitance (≈1–10 µF cm^−2^) that surpass that of field‐effect transistors by several orders of magnitude.^[^ ^22^ ^]^ Thus, EGST reports excellent gate‐to‐channel coupling efficiency.^[^ ^23^ ^]^ The simple structure of EGST enables its compatibility with various channel materials such as Si,^[^ ^24^ ^]^ metal oxides,^[^ ^25^ ^]^ organic semiconductors,^[^ ^26^ ^]^ and perovskite oxides.^[^ ^27^ ^]^ Among these, 2D transition metal dichalcogenide (TMDC) materials, particularly molybdenum disulfide (MoS_2_), have emerged as ideal candidates for EGST channels. 2D materials offer high gate modulation efficiency owing to their atomically thin nature as well as a high electron density when scaled down to atomic thickness, which is advantageous for EDL formation.^[^ ^29^ ^]^ MoS_2_, having large‐area growth technology and high process maturity compared to the other 2D materials, possesses outstanding electrical properties, including an adjustable band gap (1.2–1.8 eV), high electron mobilities (>100 cm^2^ V^−1^ s^−1^), and high on/off current ratio (>10^7^). Consequently, it has been studied extensively as an EGST channel in combination with various liquid electrolytes.^[^ ^30^, ^31^ ^]^ However, EGSTs based on liquid electrolytes face pivotal limitations, such as process incompatibility, high‐density synapse demonstrations, and thickness scaling limitations, owing to the power consumption induced by the leakage current. Therefore, it is imperative to explore EGSTs based on solid electrolytes that are compatible with MoS_2_ channels.

In this study, we demonstrate an ultrathin all‐solid‐state EGST, which incorporates an atomically thin MoS_2_ channel and a nanoscale polymer electrolyte, called MoS_2_‐EGST. Unlike previously reported EGSTs with mechanically exfoliated TMDC channels, MoS_2_ channels were grown via chemical vapor deposition (CVD) to enable large‐scale artificial synapse demonstrations. To deposit the ultrathin electrolyte layer for MoS_2_‐EGST, we employed an initiated chemical vapor deposition (iCVD) process, which allowed us to achieve a uniform and controllable thickness at the nanoscale for the solid organic‐inorganic hybrid electrolyte film. The iCVD process facilitated the synthesis of a solid electrolyte film with a thickness of 25 nm. This solid electrolyte, containing metal–OH functional groups, served the dual purpose of functioning as a gate insulator while supplying H^+^ cations. By precisely controlling the mobile ion content through a solvent‐free radical reaction‐based iCVD process, we could tune the properties of the organic‐inorganic hybrid electrolyte. Increasing the proton content expanded the switching window of MoS_2_‐EGST while achieving excellent electrical characteristics, including a digital switching window of 10^9^, an analog on/off ratio of 15, and a linear analog conductance update of 7 bits, maintaining the low switching energy of 200 fJ/spike. To evaluate the proposed MoS_2_‐EGST at the system level, we investigated the device‐to‐device (D2D) conductance update variation and performed device‐to‐system‐level simulations for MNIST digit recognition. We achieved an excellent recognition rate of 93.4% in these simulations, demonstrating the potential of MoS_2_‐EGST for practical applications.

## 2 Results and Discussion

**Figure 1:**
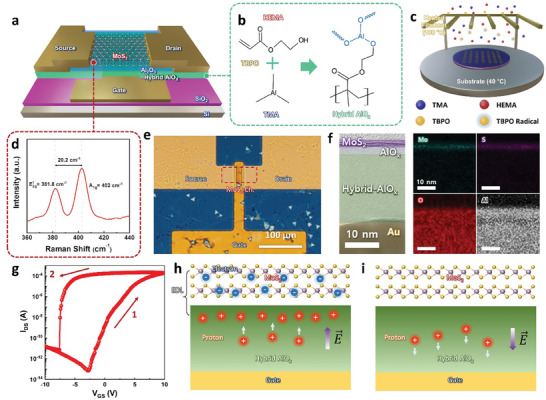
a) Description of the 3D image of the fabricated MoS_2_‐EGST. b) Schematic of the synthesis process of the Al hybrid electrolyte film. c) Schematic illustration of the iCVD process composed of heated filament, initiator, and monomers. d) Raman spectrum of the synthesized MoS_2_ film. e) Optical microscopy (OM) image of the fabricated MoS_2_‐EGST device. f) High resolution transmission electron microscopy (TEM) analysis of the MoS_2_‐EGST. Left panel is high resolution TEM image of the MoS_2_‐EGST device. Right panel is energy‐dispersive X‐ray spectroscopy (EDS) mapping of Mo‐L, S‐K, O‐K, and Al‐K of the interface of the MoS_2_ channel and electrolyte. g) Transfer curve of the fabricated MoS_2_‐EGST. h) Schematic illustration of the movement of protons within the Al hybrid under positive bias condition and i) under negative bias condition.

**Figure 2:**
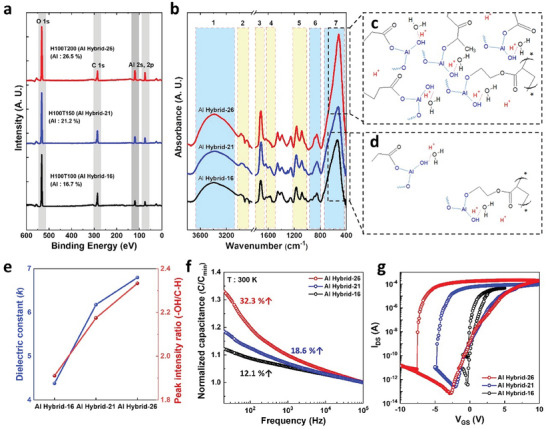
a) XPS analysis of hybrid AlO_x_ films according to different HEMA and TMA ratio. b) FTIR spectra of the three different synthesized hybrid AlO_x_ dielectric. c) Schematic explanation of the chemical composition of hybrid AlO_x_ with a HEMA‐to‐TMA ratio of 100:200 and d) that of the chemical composition of hybrid AlO_x_ with a HEMA‐to‐TMA ratio of 100:100. e) Comparison of the dielectric constant and (─OH/C─H) bond intensity of hybrid AlOx films based on three different TMA and HEMA composition ratios. f) Variation of normalized capacitance with frequency for hybrid AlO_x_ films according to HEMA and TMA composition ratios. g) Transfer curve characteristics for three different compostition of hybrid AlO_x_ films.

**Figure** **1**a shows a schematic of the ultrathin MoS_2_‐EGST. On the SiO_2_/Si substrate, the gate electrode, gate insulator, channel, and source/drain were defined sequentially (Figure S1, Supporting Information). The bottom gate was thermally evaporated and a nanoscale Al hybrid gate insulator was deposited. The Al hybrid was synthesized by polymerizing trimethylaluminum (TMA) with a 2‐hydroxyethyl methacrylate (HEMA) matrix as the backbone, as shown in Figure 1b. The hydroxyl group of HEMA reacts with TMA to generate an AlO_x_ grafted hybrid insulator, and the proton of the hydroxyl group that binds aluminum (Al) acts as a mobile ion. To fabricate a solid electrolyte based on the Al hybrid, a solvent‐free iCVD process was used (Figure 1c). iCVD is a vapor deposition process for polymeric films that involves vaporizing initiators and monomers, generating radicals with heated filaments, and inducing polymerization through free radicals.^[^ ^32^ ^]^ Owing to the solvent‐free process, iCVD has the advantage of polymerizing functional copolymers. In addition, iCVD enables uniform metal‐organic hybrid copolymer films of nanoscale thickness on a wafer‐scale process.^[^ ^33^ ^]^ In addition, the Al content in the polymeric film was precisely controlled by modulating the flow rate of the monomers (**Figure** **2**). Subsequently, atomic layer deposition (ALD) grown‐Al_2_O_3_ (4 nm) was deposited on the electrolyte to inhibit proton leakage from the electrolyte. Subsequently, CVD‐grown MoS_2_ was transferred onto a gate insulator (Figure S2, Supporting Information). The CVD‐grown MoS_2_ was analyzed with the Raman spectroscopy, and it was found that ultrathin MoS_2_ was synthesized by the narrow frequency difference Δk (20.2 cm^−1^) between the A_1g_ peak (402.0 cm^−1^) and the E^1^_2g_ peak (318.8 cm^−1^) (Figure 1d). The synthesized few‐layer MoS_2_ is considered a suitable channel material for high‐performance EGST owing to its excellent gate controllability, high mobility, wide on/off ratio, and excellent robust stability based on its low thickness. Moreover, the thickness scaling limitation of conventional electrolytes and materials hinders the performance improvement of EGST; therefore, it is essential to develop nanoscale thickness solid electrolytes and atomically thin channels.

Figure 1e shows an optical microscopy image of MoS_2_‐EGST fabricated using a large‐scale demonstration‐compatible process. Through photolithography, a MoS_2_‐EGST with a bottom‐gate transistor structure was demonstrated with a channel width of 40 µm and a channel length of 10 µm. The solid polymer electrolyte deposited through the iCVD process exhibited excellent uniformity and chemical stability, enabling compatibility with photolithography and allowing the implementation of high‐density artificial synapses. Note that, though the pHEMA film was soluble in solvents such as acetone and isopropyl alcohol, the AlO_x_ component in the hybrid copolymer film offers crosslinking by incorporating in the polymer matrix to form a chemically stable film (See Figure S3, Supporting Information). Cross‐sectional transmission electron microscopy (TEM) was conducted to identify the channel and gate dielectric of MoS_2_‐EGST (Figure 1f). The high‐resolution TEM image clearly confirmed that the ultrathin MoS_2_‐EGST was composed of two layers of MoS_2_ channels, and the nanoscale gate dielectric was composed of Al_2_O_3_ and an Al hybrid. Note that the scalability of the EGST system is limited by the conductive electrolyte requiring thick vertical dimension to prevent noise in synaptic plasticity, but nanoscale gate dielectric with limited ion conduction enables further scaling of the MoS_2_‐EGST device. Energy‐dispersive X‐ray spectrometry (EDS) analysis revealed a uniform distribution of Al in the Al hybrid achieved through the iCVD process, which is advantageous for the generation of random copolymers based on free radicals. Figure 1g shows the transfer curves of the MoS_2_‐EGST with a gate voltage (V_GS_) dual sweep from −10 to 10 V and a drain voltage (V_DS_) of 1 V, which were measured under a vacuum (<10^−2^ Torr) chamber to prevent unintentional channel doping due to ambient air adsorption. The device exhibited counterclockwise hysteresis with a higher threshold voltage (V_th_) in the forward sweep and a lower V_th_ in the backward sweep, showing characteristics similar to those of memory devices with an increasing hysteresis range as the measurement range of the gate voltage increased (Figure S4, Supporting Information). Using a solid electrolyte with insulating properties, the device showed low gate leakage and suppressed doping of the MoS_2_ channel by the solution, resulting in an excellent on/off ratio (>10^9^) and low subthreshold swing (SS_backward_ = 59 mV dec^−1^). Note that the low subthreshold swing is due to the generation of EDL capacitance resulting from the movement of positive ions in the electrolyte. Figure 1h,i illustrates the switching mechanism of the EDL capacitance of MoS_2_‐EGST. Once a positive bias is applied to the gate, the positive ions in the electrolyte move toward the channel, creating a positively charged layer. The accumulation of electrons in the MoS_2_ channel forms a negatively charged layer, thus establishing the EDL (Figure 1h). However, when a negative bias is applied to the gate, the positive ions near the channel move away from the channel, not only reducing the EDL by the gate bias, but also further collapsing the EDL as it progressively weakens, resulting in a decrease in the channel conductance exhibiting an SS_backward_ lower than the subthreshold swing limit (SS_limit_ = 60 mV dec^−1^ at 300 K).

The Al hybrid electrolyte synthesized using iCVD for EGST devices offers the advantage of an adjustable proton content. The composition of the Al hybrid electrolyte was precisely controlled by varying the flow rates of TMA and HEMA. In this study, three polymeric films with HEMA:TMA flow rate ratios of 100:200 (H100T200), 100:150 (H100T150), and 100:100 (H100T100) were synthesized and analyzed. X‐ray photoelectron spectroscopy (XPS) was employed to analyze the atomic composition and chemical bonding of the synthesized films (Figure 2a). The Al content increased with the TMA flow rate, with values of 16.7%, 21.2%, and 26.5% for H100T100 (Al Hybrid‐16), H100T150 (Al Hybrid‐21), and H100T200 (Al Hybrid‐26), respectively. To investigate the chemical bonding of each major element, high‐resolution scans of the C1s, O1s, and Al2p peaks were analyzed (Figure S5, Supporting Information). The Al 2p peak binding energy exhibited a negative shift as the TMA content increased. Considering that the Al 2p peak is composed of Al_2_O_3_ (74.1 eV) and Al_2_(OH)_3_ (74.8 eV),^[^ ^34^, ^35^ ^]^ an increase in the TMA content not only led to an increase in the Al content but also an increase in the proportion of Al─OH bonds.

The chemical properties of these films were further validated using Fourier transform infrared spectroscopy (FTIR) analysis (Figure 2b). The characteristic peaks corresponding to HEMA appeared at 3400, 2890, 1720, 1483, 1210, and 1079 cm^−1^, while the characteristic peaks corresponding to AlO_x_ appeared at 850 and 550 cm^−1^.^[^ ^36^, ^37^, ^38^ ^]^ Note that the characteristic peaks at 3400, 850, and 550 cm^−1^ increase with increasing TMA content. Based on the FTIR analysis, schematic images of the films with the highest (Al Hybrid‐26) and lowest TMA contents (Al Hybrid‐16) are shown in Figure 2c,d. The Al Hybrid‐26 film, which contains a higher TMA content, exhibits a structure in which multiple AlO_x_ moieties are grafted with hydroxyl groups within the HEMA matrix, and additional hydroxyl groups are connected to Al, resulting in a higher density of the hydroxyl groups. Notably, the ability to control the density of the hydroxyl groups in the electrolyte is a significant advantage for the fabrication of EGST. As the EDL formation in EGST is influenced by the mobile ion mobility and density, the control of hydroxyl groups enables the regulation of synaptic characteristics. X‐ray photoelectron spectroscopy and FTIR analyses confirmed that the iCVD process allowed precise control of the AlO_x_ content within the HEMA matrix by adjusting the TMA flow rate. Moreover, by controlling the AlO_x_ content, the hydroxyl group concentration can be modulated. Note that, the electrolyte containing proton usually exhibits chemical instability toward air and water, which can penetrate into the electrolyte and change its proton conductivities. Even though we show that water can hardly penetrate the hybrid polymer electrolyte (Figure S3, Supporting Information), we suggest adopting additional passivation layers to enhance the chemical stability of the electrolyte for the practical use of MoS_2_‐ESGT.

The capacitance characteristics of the Al hybrid electrolyte films were analyzed to investigate the characteristics of electrolytes with different hydroxyl group densities. Figure 2e shows the relation between measured relative dielectric constants and OH/C–H peak ratio. Dielectric constants were calculated based on the capacitance values at a temperature of 303 K and frequency of 20 Hz and measured dielectric thickness of the three synthesized films (H100T100, H100T150, and H100T200) in Figure S6 (Supporting Information). The peak intensity ratios of the hydroxyl peak (3400cm^−1^) to the C–H bending peak (1458cm^−1^) were calculated based on our FTIR analysis in Figure 2b.^[^ ^33^ ^]^ The films with a higher proportion of grafted‐AlO_x_ within the HEMA matrix exhibited not only a higher dielectric constant, which enabled better control of the channel, but also a higher proportion of hydroxyl groups. The increase in capacitance owing to the presence of hydroxyl groups is shown in Figure 2f. It can be observed that the capacitance at 20 Hz in Al Hybrid‐16, Al Hybrid‐21, and Al Hybrid‐26 films increased by 12.1%, 18.6%, and 32.3%, respectively, compared to the capacitance at 100 kHz. This increase in capacitance is attributed to the polarization of the insulator within the film as well as the effect of proton polarization induced by the low‐frequency alternating voltage.^[^ ^39^ ^]^ Consequently, films with a higher density of hydroxyl groups provide greater capacitance enhancement by supplying a larger number of mobile ions. The increase in capacitance due to the presence of abundant protons leads to the generation of an EDL by inducing the accumulation of protons in the channel under a positive bias. The transfer curves of MoS_2_‐EGT with different hydroxyl group densities are shown in Figure 2g. The higher density of the hydroxyl groups in the Al hybrid resulted in a larger hysteresis effect owing to the formation of a high‐density EDL, which induced a higher on‐current and mobility (Figure S7, Supporting Information).

**Figure 3:**
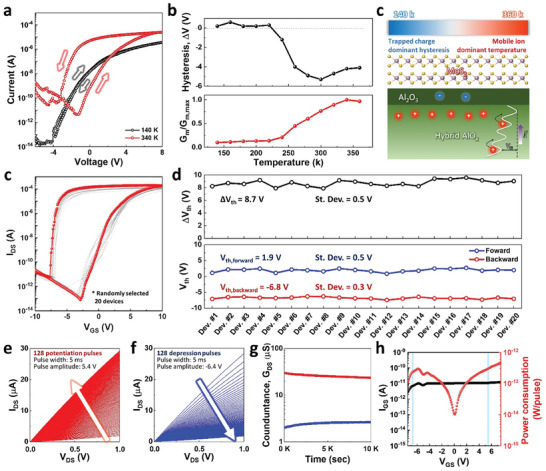
a) Transfer curve characteristics of MoS_2_‐EGST devices according to the temperature of 140 and 340 k. b) Variation of hysteresis and transconductance (G_m_) values according to different temperature region and c) the schematic representation of the behavior of excess protons in hybrid AlO_x_ dielectric under different temperature range. d) Transfer curve characteristics of 20 randomly selected devices of MoS_2_‐EGST. e) Comparison of hysteresis and threshold voltage characteristics of the 20 random devices. f) Verification of potentiation characteristics and g) depression characteristics of MoS_2_‐EGST device. g) Retention characteristics of MoS_2_‐EGST devices in full potentiation state and full depression state for 10^5^ s. h) Gate leakage current and power consumption characteristics of MoS_2_‐EGST as a function of gate voltage.

We fabricated MoS_2_‐EGT using an Al Hybrid‐26 thin film with a high dielectric constant and abundant hydroxyl groups and confirmed its electrical properties. From our previous work, we confirm that 28% of Al content represents the saturated maximum Al percentage that can be integrated into the polymer matrix.^[^ ^43^ ^]^ The transfer curve of the fabricated MoS_2_‐EGT was analyzed at various temperatures to observe the movement of ions and charges (**Figure** **3**a). The transfer curves at 140 and 220 K show no noticeable hysteresis, whereas the curves at 260 and 340 K exhibit significant counterclockwise hysteresis (Figure S8, Supporting Information). Furthermore, a significant increase in the on‐current is observed as the temperature increases. Figure 3b represents the variations of hysteresis (ΔV_TH_) and maximum transconductance (G_m_) at each applied temperature condition estimated from the graph in Figure S9 (Supporting Information). Below 220 K, the curve exhibits a small clockwise hysteresis with a lower maximum G_m_, whereas above 240 K, it shows a counterclockwise hysteresis with an increasing maximum G_m_. This indicates that at low temperatures, the hysteresis is dominated by electron trapping owing to the limited proton mobility. However, as the temperature increases, proton movement is enhanced, resulting in a counterclockwise hysteresis (Figure 3c). As proton conduction in an Al hybrid matrix is governed by the Grotthuss mechanism,^[^ ^40^, ^41^ ^]^ protons exhibit a higher hopping probability as the temperature increases. Moreover, the maximum transconductance increases with temperature owing to the movement of mobile ions. In the case of MoS_2_‐EGT, the increased maximum transconductance was attributed to the increase in capacitance owing to the enhanced movement of protons at high temperatures (Figure S10, Supporting Information).

Note that the demonstration of artificial synapses in a high‐density crossbar array is essential and requires the deposition of channel and gate insulator materials that are compatible with large‐area fabrication. The MoS_2_‐EGTs utilized the vapor phase to synthesize channels and insulators, enabling the fabrication of transistors in a large‐area region; all solid‐state devices were compatible with the photolithography process. Among the transistors, Figure 3c shows the transfer curves of 20 randomly selected MoS_2_‐EGTs with a V_GS_ range from −10 to 10 V and a drain voltage of 1 V. Owing to the excellent mobility and low defect density of the CVD‐grown MoS_2_, MoS_2_‐EGTs achieved a wide on/off ratio over 10^9^, exhibiting considerable hysteresis in the counterclockwise direction memory switching. Notably, while 2D materials‐based EGTs mostly focus on device‐level demonstrations using flake‐based channels (**Table** **1**), this study reports MoS_2_‐EGTs in a large‐scale domain with CVD‐grown MoS_2_ channels and iCVD‐grown electrolytes. Figure 3d shows a statistical analysis of the V_th_ and V_th_ shifts in the memory switching of the MoS_2_‐EGTs. The V_th_ was determined at a gate voltage corresponding to a drain current of 100 nA and a drain voltage of 1 V. The average forward threshold voltage (V_th,forward_) of 1.9 V and backward threshold voltage (V_th,backward_) of −6.8 V were obtained, with standard deviations of 0.5 and 0.3 V, respectively. A narrow V_th_ distribution achieved a uniform hysteresis window of 8.7 V, with a standard deviation of 0.5 V.

**Table 1:** Summary of EGSTs with various channel materials.

Channel (Active)	Electrolyte	Channel process/Thickness [nm]	Electrolyte process/Thickness [nm]	Channel length [µm]/Channel width [µm]	Mobile ion	# of bits	Pulse width	HDR	Retention time/states	Switching Energy [fJ/sptike]	Electrolyte tunable	Large Area Process compatible
MoS_2_ (This work)	pHEMA‐AlO_x_ (20 nm)	CVD/< 2	iCVD/25	10/40	H^+^	7	5 ms	≈15	1000 s/11	200	Yes	Yes
WSe_2_^[^ ^44^ ^]^	Ion gel (LiClO_4_/PEO)	Mechanical exfoliation/> 5	Mixture drop/–	1/3	Li^+^	6 (60)	50 ms	2.3		30	No	No
α‐MoO_3_^[^ ^45^ ^]^	Ion gel (LiClO_4_/PEO)	Mechanical exfoliation/∼ 20	Dip‐coating/–	10/5	Li^+^	5 (50)	10 ms	1.36		1800	No	No
α‐MoO_3_^[^ ^46^ ^]^	Ionic liquid	Mechanical exfoliation/< 20	Liquid drop/–	5/3	H^+^	5 (50)	1 ms	1.62		9600	No	No
SmNiO_3_^[^ ^47^ ^]^	Ionic liquid	Sputtering/≈5	Liquid drop/–	–	O^2−^	>7	10 ms	<1		–	No	No
IGZO^[^ ^48^ ^]^	HfO_2_	Sputtering/20	Sputtering/250	–	H^+^	100 (6)	1 ms	–	200s/2	40290	Yes	Yes
W^[^ ^49^ ^]^	WO_3‐x_ (80 nm)	Sputtering/5	Sputtering/∼ 100	–	O^2−^	6	10 ms			–	Yes	Yes
Si^[^ ^24^ ^]^	pEGDMA	Commercial Si wafer/–	iCVD/20	7/50	H^+^	7	10 ms	≈3	5000 s/5	20	No	Yes

In the programmed state of MoS_2_‐EGT, MoS_2_ had a high electron density owing to the accumulation of protons near the channel. Furthermore, we report a gradually adjustable channel conductance achieved by modulating the proton density using consecutive gate voltage pulses. Figure 3e,f depicts the changing output curves as potentiation and depression (P/D) occur with pulses applied to the gate. Each of the output curves was attained by measuring drain current (I_DS_) with respect to the V_DS_ after applying a single gate pulse. The set of P/D output curves consists of 128 pairs of *I*_D_‐*V*_D_ read operation and gate programming pulses with a width of 5 ms and amplitudes of 5.4 and −6.4 V, respectively. The separable drain current indicates the tunability of the analog conductance to implement artificial synapses. Additionally, the linear relationship between V_DS_ and the I_DS_ enables analog MAC operations with analog input voltages based on Ohm's law. The solid electrolyte enables the maintenance of the nonvolatile EDL, and the retention of the maximum potentiation and depression states is shown in Figure 3g. The MoS_2_‐EGTs demonstrated separable retention even after 10^4^ s without the need for additional barrier layers or channel doping based on the Grotthuss mechanism. Moreover, the solid electrolyte of the MoS_2_‐EGTs exhibited limited ion conduction, resulting in low gate leakage currents (Figure 3h). We can calculate the switching energy by multiplying the programming voltage by the amount of charge, which can be estimated by multiplying the gate leakage current by the pulse width of 5 ms, for a weight update. With low gate leakage currents, MoS_2_‐EGTs exhibit a low switching energy equivalent to 200 fJ (Table 1), and we expect further device scaling may lead to lower switching energy by lowering the gate leakage current.

**Figure 4:**
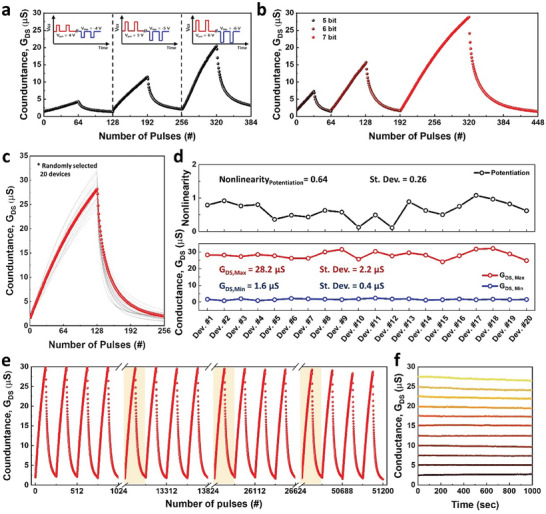
PD curve of MoS_2_‐EGST (H100T150) with a) different pulse amplitude and b) different pulse number. c) The PD curve characteristics of 20 randomly selected MoS_2_‐EGSTs. d) Comparison of nonlinearity value at potentiation condition and the conductance states at full potentiation and full depression states of the selected MoS_2_‐EGSTs. e) Endurance characteristics of MoS_2_‐EGST devices for 51200 pulses. f) Stable retention characteristics of 11 different trained states of MoS_2_‐EGST synaptic devices.

The artificial synapse to accelerate the MAC operation should mimic the long‐term plasticity of the biological synapse. An artificial synapse should store synaptic weight in a non‐volatile manner, which can also be modulated, to conduct multiplication operations with the input data. Non‐volatile MoS_2_‐EGST can be used as an artificial synapse by gradual conductance modulation of the channel through gate voltage pulses. Channel conductance is determined by the distribution of protons in the electrolyte, which is governed by the amplitude and number of gate voltage pulses. **Figure** **4**a illustrates the conductance updates under 64 pulses of P/D with the pulse widths of 5 ms and pulse amplitudes of ±4, 5, and 6 V. With the increasing potentiation gate voltage of 4, 5, and 6 V (even with the same number of pulses), the maximum drain conductance increases to 4.3, 11.5, and 20.4 µS, respectively, while the minimum conductance decreases to 1.4, 2.1, and 3.2 µS, respectively. Utilizing high programming voltages allows a wide analog conductance range, enabling the stable implementation of multiple conductance states. Furthermore, MoS_2_‐EGST demonstrated a tunable conductance update region based on the number of pulses (Figure 4b). Under the same pulse conditions (potentiation: 5.4 V/5 ms, depression: −6.4 V/5 ms), as the number of potentiation pulses increases to 32, 64, and 128, the maximum conductance increases to 7.3, 15.4, and 28.8 µS, respectively. This analog‐adjustable conductance range can also be observed through the synthesis of electrolytes that allow control of the cation concentration (Figure S11, Supporting Information). Notably, the analog conductance range can be adjusted while maintaining linear potentiation. The linear conductance update is achieved by the excellent gate controllability of the ultrathin MoS_2_, making MoS_2_‐EGST suitable for artificial synapses. The relatively slow switching speed of MoS_2_‐ESGT can be improved through further studies by enhancing the proton conductivity of the electrolyte.

Nevertheless, reliability and variability issues, such as cycle‐to‐cycle variation, D2D variation, and retention impede the practical implementation of energy‐efficient neuromorphic computing. MoS_2_‐EGST was expected to address these issues, and the reliability characteristics of MoS_2_‐EGST were evaluated. To examine the spatial variation of MoS_2_‐EGSTs, we investigated the P/D curves (potentiation: 5.4 V/5 ms, depression: −6.4 V/5 ms) in 20 randomly selected MoS_2_‐EGTs in Figure 3c (Figure 4c; Figure S12, Supporting Information). MoS_2_‐EGST exhibits linear conductance updates and maintains a uniform conductance range. The nonuniform conductance ranges of artificial synapses, owing to device variations, result in biased weight storage, which leads to a decrease in recognition accuracy in neuromorphic systems.^[^ ^42^ ^]^ For EGST fabrication, ensuring uniform channel conductance is challenging in solution‐based synthesized and flake‐based layered material channels. CVD‐grown MoS_2_, which can provide uniform channel conductance, is suitable for large‐scale demonstration of artificial synapses. In addition, the iCVD process enables uniform random copolymer fabrication on the wafer scale^[^ ^33^, ^43^ ^]^ providing reliable conductance updates in all devices based on a uniform proton distribution. As a result, MoS_2_‐EGST exhibits an average nonlinearity of 0.64 with a standard deviation of 0.26, an average G_DS,Max_ of 28.2 µS with a standard deviation of 2.2 µS, and an average G_DS,Min_ of 1.6 µS with a standard deviation of 0.2 µS (Figure 4g). MoS_2_‐EGST also demonstrates excellent spatial variation, as evaluated by cycle endurance measurements (Figure 4e). MoS_2_‐EGST maintains linear conductance updates even after 5×10^4^ switching cycles, exhibiting an insignificant decrease of 7% in maximum conductance (from 30.3 to 2.81 µS) due to the fatigue. This indicates that the HEMA matrix‐based electrolyte enables nondestructive proton conduction and prevents the diffusion of protons out of the electrolyte. Additionally, Figure 4f confirms the separable analog conductance states for over 10^3^ s with 2.5 µS intervals. The limited ion movement within the HEMA matrix‐based electrolyte allows for nonvolatile EGST where the retention property rather starts to degrade as temperature increased up to 350 K as the proton‐based EDL phenomenon is susceptible to moisture reduction and breakdown of polymers at high temperatures (Figure S13, Supporting Information). The synaptic performances of MoS_2_‐EGST using CVD‐grown MoS_2_ and hybrid electrolytes are compared in Table 1 and Table S1 (Supporting Information).

**Figure 5:**
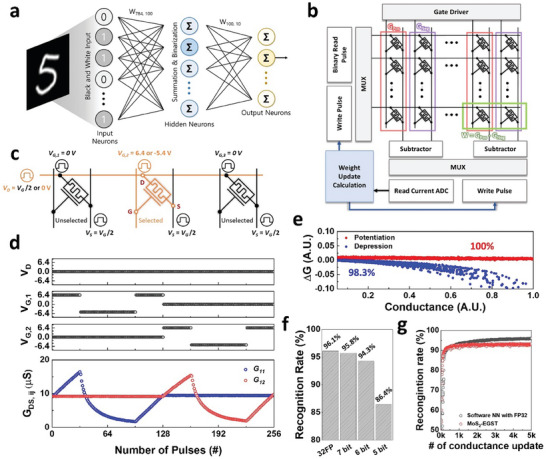
Array performance simulation of the MoS_2_‐EGST. a) Two‐layer artificial neural network with 784 input neurons, 100 hidden neurons, and 10 output neurons the MNIST handwritten digit recognition. b) Schematic of the circuit block diagram of the MoS_2_‐EGST array including peripherals. Parallel update programming scheme of the MoS_2_‐EGST: (c) schematic illustration and d) half‐voltage programming. e) Device‐to‐device aware weight update accuracy of the MoS_2_‐EGST. f) Inference performance of MoS_2_‐EGST with the software‐trained weight. g) MNIST handwritten digit recognition accuracy for the training of the MoS_2_‐EGST.

The performance of the MoS_2_‐EGST‐based neuromorphic system was evaluated through device‐to‐system‐level simulations. A 3‐layer multi‐layer perceptron based on error backpropagation learning, consisting of 784 inputs and 100 hidden neurons, was used for MNIST handwritten digit recognition with 60 000 training data points and 10 000 test data points, as shown in **Figure** **5**a. Figure 5b shows a block diagram of the circuit implementation of MoS_2_‐EGST‐based neuromorphic synapse array. Differential of the two MoS_2_‐EGSTs was used to map a single synapse weight to demonstrate a negative synaptic weight. The peripheral circuits were designed for a pulse‐based parallel programming scheme, as shown in Figure 5c. The programming device was selected using drain drivers connected to the row and gate drivers connected to the column. It is essential to introduce the half‐voltage scheme to program a single cell in 1T synapse array configuration (See Figure S14, Supporting Information). Since a synapse array must have input vector rows and output vector columns, a transistor‐based synapse array inevitably intersects the source line that corresponds to the output column, and the drain line that corresponds to the input row. Thereby, either of the source line and drain line must be placed parallel to the gate line, which makes it impossible to program a single cell with the ground scheme, as shown in Figure S14a (Supporting Information). The accessed device received a voltage of V_GD_ = V_prog_ and V_GS_ = V_prog_/2 that enables conductance update to the selected cell, while unselected devices receive a half‐programming voltage of V_GD_ = 0 V and V_GS_ = −V_prog_/2 that cannot change the conductance of the unselected cell. Figure 5d demonstrates the conductance update characteristics of two MoS_2_‐EGST devices programmed using the half‐voltage scheme. The unselected device maintained its conductance state at the half‐programming voltage, whereas the selected device successfully modulated its conductance. The half‐voltage programming scheme allows synaptic device programming without additional access devices, thereby enabling the implementation of high‐density synapse arrays, as shown in Figure 5b. Each synaptic device was individually modeled with the weight‐update nonlinearity assigned by Monte Carlo sampling using the spatial variation data in Figure 4c, showing 100% potentiation accuracy and 98.3% depression accuracy (Figure 5e). Note that the low write stochasticity of the MoS_2_‐EGST enables conductance mapping to the target synapse weight only with few programming pulses, which makes the EGST system a competitive artificial synaptic device even with relatively slow switching. The inference and training performances of the MoS_2_‐EGST‐based neuromorphic system with excellent synaptic characteristics are shown in Figure 5f,g. For the inference performance evaluation, software‐trained synapse weights were quantized and mapped to MoS_2_‐EGST, as shown in Figure 5f. The system achieved a recognition accuracy of 96.1% with weights trained in 32‐bit floating point (32FP), and performance degradations of 0.3%, 1.8%, and 9.7% were observed when the MoS_2_‐EGST was quantized to 7‐bit, 6‐bit, and 5‐bit, respectively. Furthermore, simulations were performed to evaluate the training performance of MoS_2_‐EGST (Figure 5g), which showed a performance degradation of 2.6% compared to ideal synaptic devices based on 32FP, achieving a recognition rate of 96%.

## 3 Conclusion

The proposed ultrathin MoS_2_‐EGST utilizes CVD‐grown MoS_2_ channels and an iCVD‐based Al hybrid as a proton electrolyte to achieve an ideal artificial synapse. The iCVD‐process‐based Al hybrid electrolyte provides a nanoscale solid‐state thin film, enabling all‐solid‐state EGSTs and high‐density artificial synaptic arrays. In addition, it allows for the implementation of synapses with a wide range of conductances by providing an abundant and adjustable proton density. Additionally, high‐quality CVD‐grown MoS_2_ enables highly uniform MoS_2_‐EGSTs with excellent gate controllability and high mobility by the EDL. Consequently, the demonstrated MoS_2_‐EGSTs achieve a digital switching window of 10^9^, state retention of 10^3^, low energy consumption of 200 fJ/spike, an analog on/off ratio of 15, and linear analog conductance updates of 7 bits. Furthermore, a neuromorphic system based on the MoS_2_‐EGST synaptic array was proposed, and system‐level simulations confirmed its inference and training performances for MNIST digit recognition of 95.8% and 93.2%, respectively. Our research not only proposes suitable channel materials for demonstrating EGST‐based synaptic arrays, but also opens up a pathway for the fabrication of high‐density and high‐performance synaptic devices using novel iCVD‐based electrolytes to overcome the scaling limitation of EGST.

## 4 Experimental Section

### Polymeric Film Deposition via iCVD

A hybrid AlOx dielectric film was synthesized using an iCVD reactor, where HEMA (99%, Aldrich, USA), TMA (99.99%, UP Chemical, Korea), and TBPO (99%, Aldrich, USA) were vaporized and introduced without additional purification. The temperatures of HEMA, TMA, and TBPO were adjusted to 60, 30, and 30 °C, respectively, to ensure a consistent source injection line pressure. The source injection line pressures of HEMA and TBPO were set to 100 mTorr, while the source injection line pressure rate of TMA was varied from 100 to 200 mTorr to synthesize organic/inorganic hybrid films with different Al compositions. The chamber pressure and substrate temperature were maintained at 300 mTorr and 40 °C, respectively. Polymerization was initiated by setting the filament temperature to 180 °C.

### MoS_2_ Synthesis Process

The MoS_2_ thin film was synthesized using atmospheric‐pressure CVD (APCVD) process in a 2 inch diameter quartz tube. Throughout the deposition process, a high‐purity Ar gas flow of 120 sccm served as the carrier gas at atmospheric pressure (750 Torr). In the typical growth procedure, 160 mg of sulfur powder was placed upstream of the furnace, while 2.6–2.8 mg of molybdenum trioxide (MoO_3_) powder was positioned downstream of the furnace along with the SiO2/Si receiving substrate. To facilitate growth, perylene‐3,4,9,10‐tetracarboxylic acid tetrapotassium salt (PTAS) was applied as a seeding promoter on the receiving substrate. The upstream and downstream sections of the furnace were heated to 830 and 320 °C, respectively.

### Device Fabrication

A Cr/Au (8 nm/50 nm) bottom‐gate electrode was deposited onto a piranha‐cleaned Si/SiO_2_ substrate using a thermal evaporator and patterned using a lift‐off process. Subsequently, a hybrid AlO_x_ dielectric layer with a thickness of 25 nm that served as an electrolyte reservoir, was deposited on the bottom gate via iCVD. Subsequently, an Al_2_O_3_ barrier layer with a thickness of 4 nm was deposited using ALD. To form the channel region, the synthesized powdered CVD MoS_2_ was transferred onto the Al_2_O_3_/Hybrid AlO_x_ dielectric layer and defined by photolithography and O_2_ plasma etching (O_2_ 100 sccm, 100 W). Finally, the source/drain electrode was formed by depositing Cr (5 nm)/Au (50 nm) using a thermal evaporator and lift‐off process.

### Device Characterization

Keithley 4200 semiconductor parameter analyzer was used to investigate the electrical characteristics of the fabricated device. Electrical measurements were performed in a vacuum chamber. DC sweep and pulse measurements were performed using Keithley 4200, Keithley 4225‐PMU (pulse generator), and 4225‐RPM (remote amplifier/switch).

## Conflict of Interest

The authors declare no conflict of interest.

## Supporting information

Supporting Information

## Data Availability

The data that support the findings of this study are available from the corresponding author upon reasonable request.
