# Supplementary material for: Ultrathin All‐Solid‐State MoS2‐Based Electrolyte Gated Synaptic Transistor with Tunable Organic–Inorganic Hybrid Film
Source: Adv Sci (Weinh). 2024 Apr 2;11(23):2308847. doi: 10.1002/advs.202308847 (PMC11187882; doi:10.1002/advs.202308847)
Supplement: Supplementary file 1 — Supporting Information [file ADVS-11-2308847-s001.pdf]

ADVANCED SCIENCE
Open Access

Supporting Information

for *Adv. Sci.*, DOI 10.1002/advs.202308847

Ultrathin All-Solid-State MoS_2-Based Electrolyte Gated Synaptic Transistor with Tunable Organic–Inorganic Hybrid Film

Jungyeop Oh, Seohak Park, Sang Hun Lee, Sungkyu Kim, Hyeonji Lee, Changhyeon Lee, Woonggi Hong, Jun-Hwe Cha, Mingu Kang, Jun Hyup Jin, Sung Gap Im, Min Ju Kim and Sung-Yool Choi**

Copyright WILEY-VCH Verlag GmbH & Co. KGaA, 69469 Weinheim, Germany, 2016.

Supporting Information

Ultrathin All Solid State MoS_2-based Electrolyte Gated Synaptic Transistor with Tunable Nanoscale Organic-Inorganic Hybrid Electrolyte Film

Jungyeop Oh, Seohak Park, Sang Hun Lee, Sungkyu Kim, Hyeonji Lee, Changhyeon Lee, Woonggi Hong, Jun-Hwe Cha, Mingu Kang, Jun Hyup Jin, Sung Gap Im, Min Ju Kim and Sung-Yool Choi**

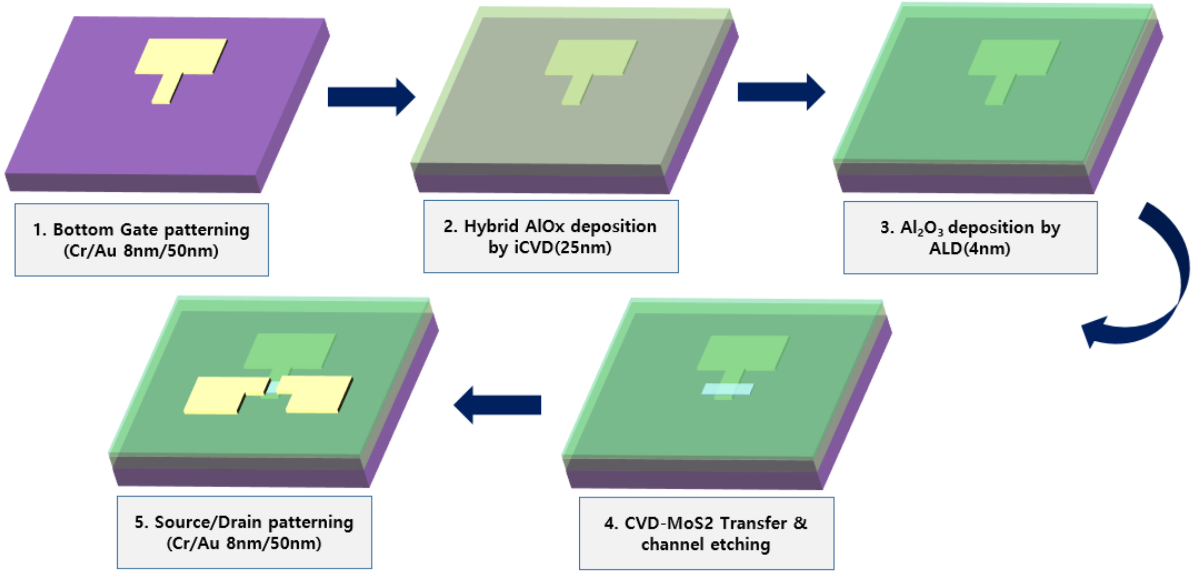

Figure S1. Schematic diagram of the fabrication process for the MoS_2-EGST device.

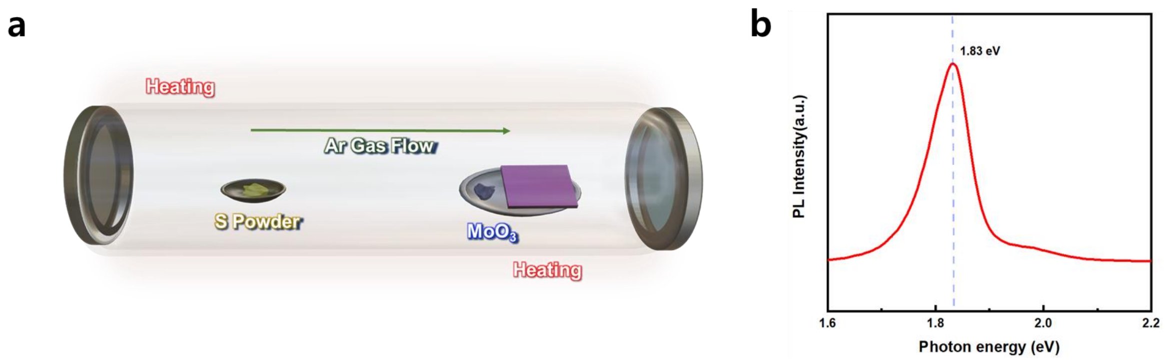

Figure S2. (a) Schematic diagram of the powder source-based APCVD synthesis process for MoS_2. (b) Photoluminescence (PL) measurement of the synthesized monolayer MoS_2.

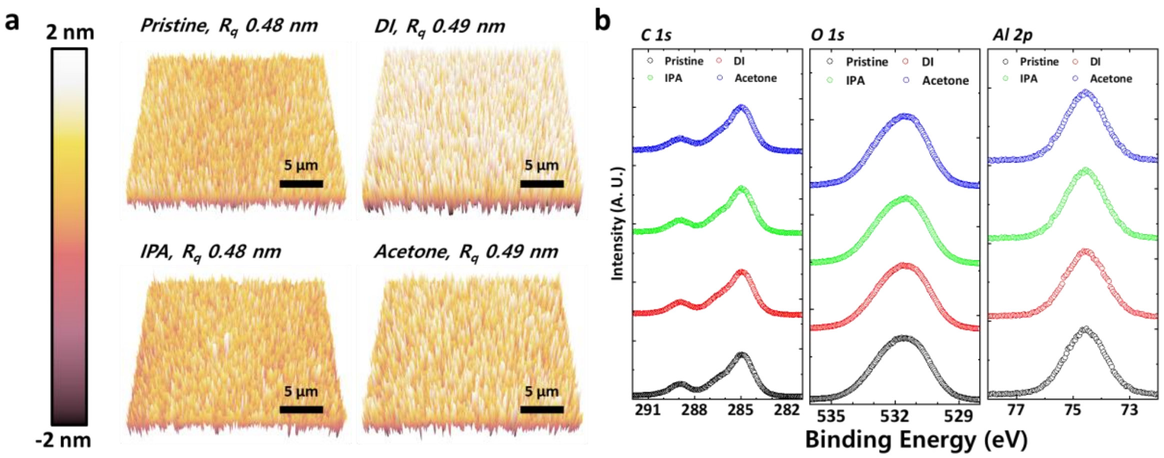

Figure S3. Chemical instability of the hybrid electrolyte under soaking various solvents. (a) atomic force microscopy surface images and (b) XPS analysis after dipping in DI water, acetone and IPA.

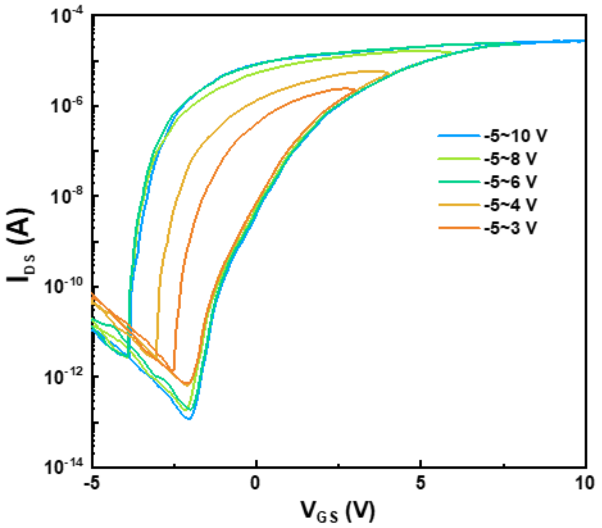

Figure S4. Variation of transfer curve characteristics of MoS_2-EGST transistor (H100T200) as a function of the gate voltage sweep range.

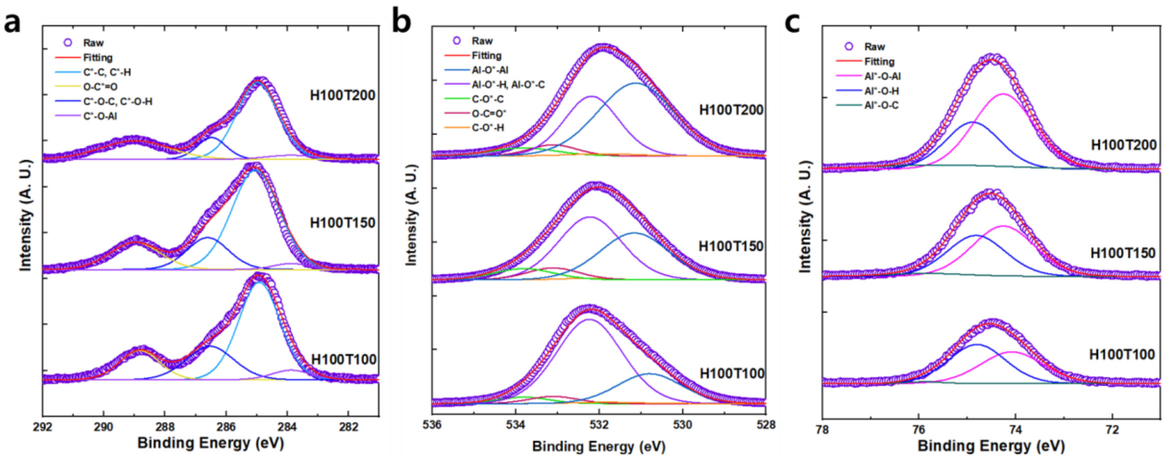

Figure S5. XPS deconvolution spectrum of the hybrid-AlO_x with different HEMA to TMA ratios of iCVD process condition. (a) C 1s, (b) O 1s, and (c) Al 2p spectra of the hybrid-AlO_x.

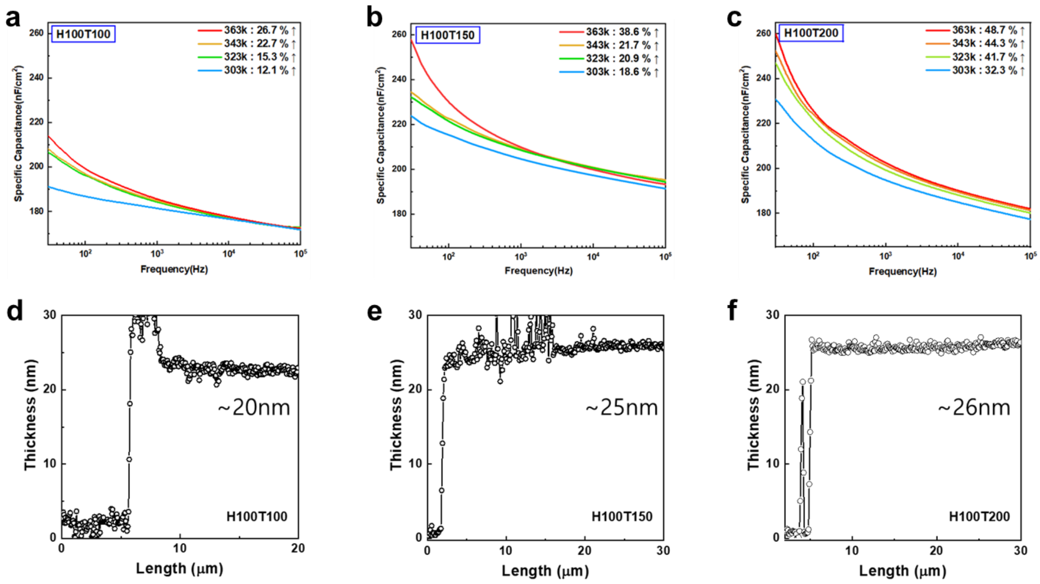

Figure S6. The variation in capacitance with frequency for Hybrid-AlO_x thin film MIM capacitors, as a function of TMA ratio and measured thickness of the three different samples using AFM. The HEMA and TMA ratios were (a) 100:100, (b) 100:150, and (c)100:200 for each case. The measured dielectric thicknesses for each sample was (d) 20nm (H100T100) (e) 25nm (H100T150) and (f) 26nm (H100T200), respectively.

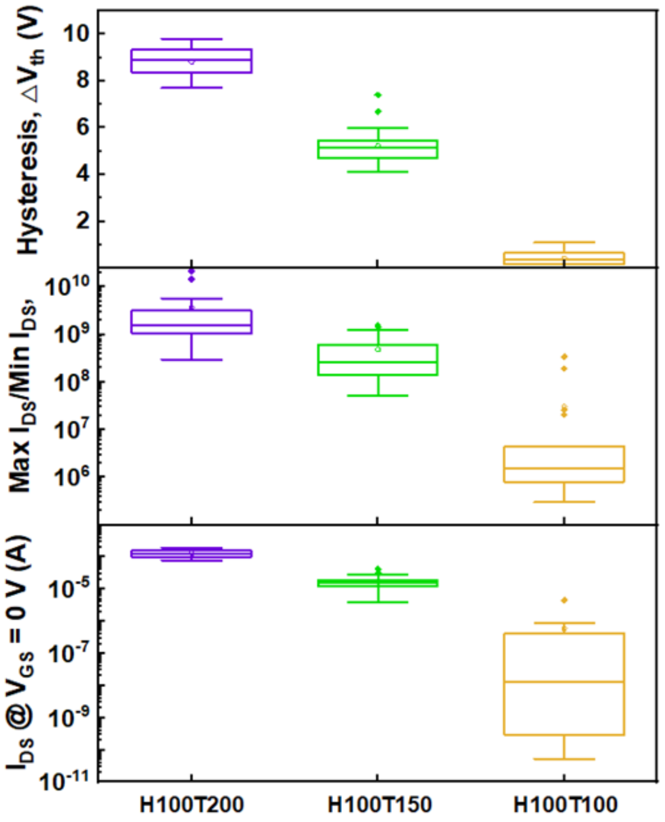

Figure S7. Comparison of the hysteresis, ratio of max drain current to minimum drain current (I_{on}/I_{off}) value and a drain current at the gate voltage of 0 V with respect to the different HEMA and TMA ratios of the hybrid-AlO_x dielectric.

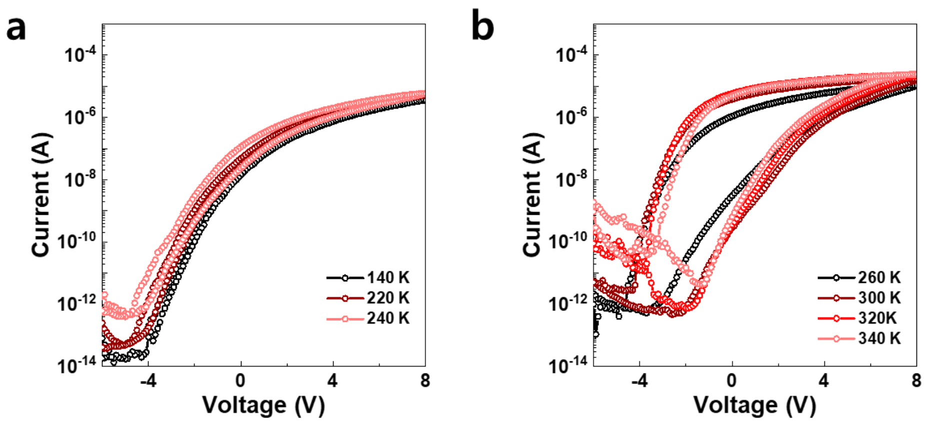

Figure S8. Transfer curve characteristics of MoS_2-EGST devices according to the temperature range from (a) 140K to 240k and (b) 260K to 340k.

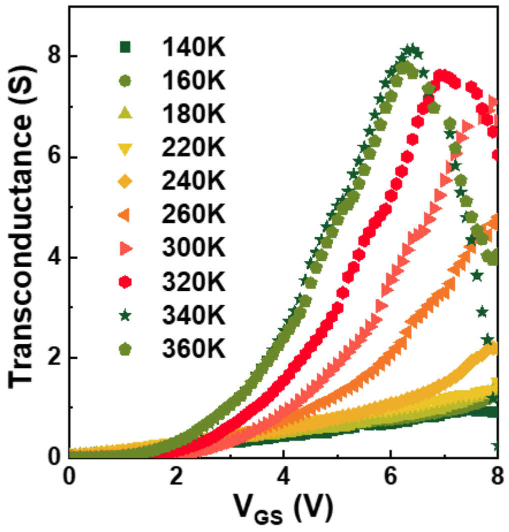

Figure S9. Variations of transconductance (G_m) characteristics of MoS_2-EGST devices according to the temperature range from 140K to 360K.

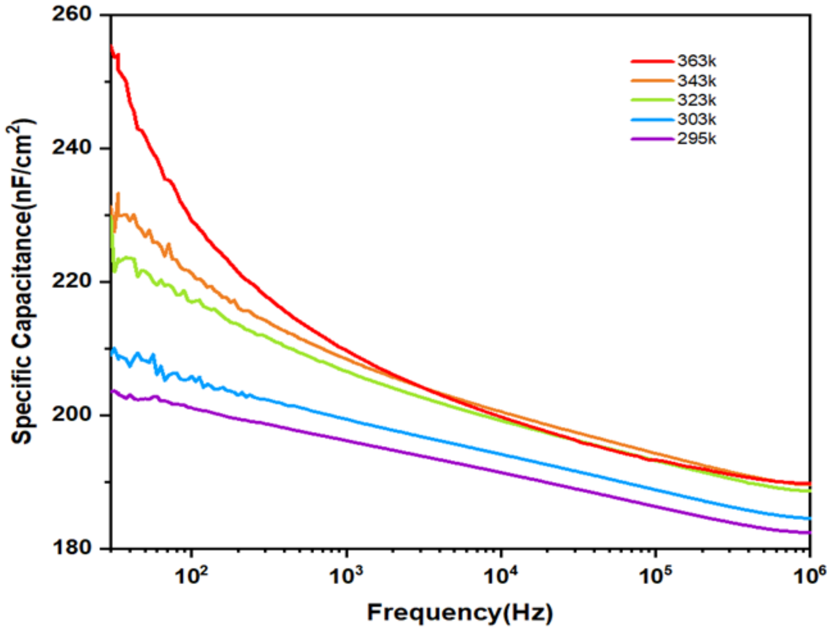

Figure S10. The frequency-capacitance characteristics of the Al Hybrid-26 MIM capacitor which were investigated over a temperature range from 295 K to 363 K

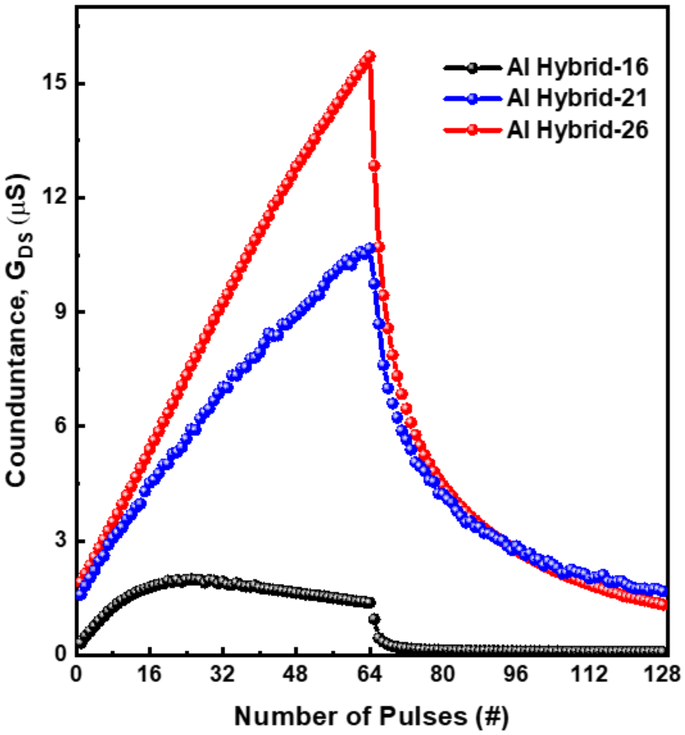

Figure S11. Comparison of PD curve characteristics in 7-bit operation of MoS_2-EGST devices based on the three different compositions ratio of hybrid-AlO_x dielectric film (Al Hybrid-16, Al Hybrid-21 and Al Hybrid-26).

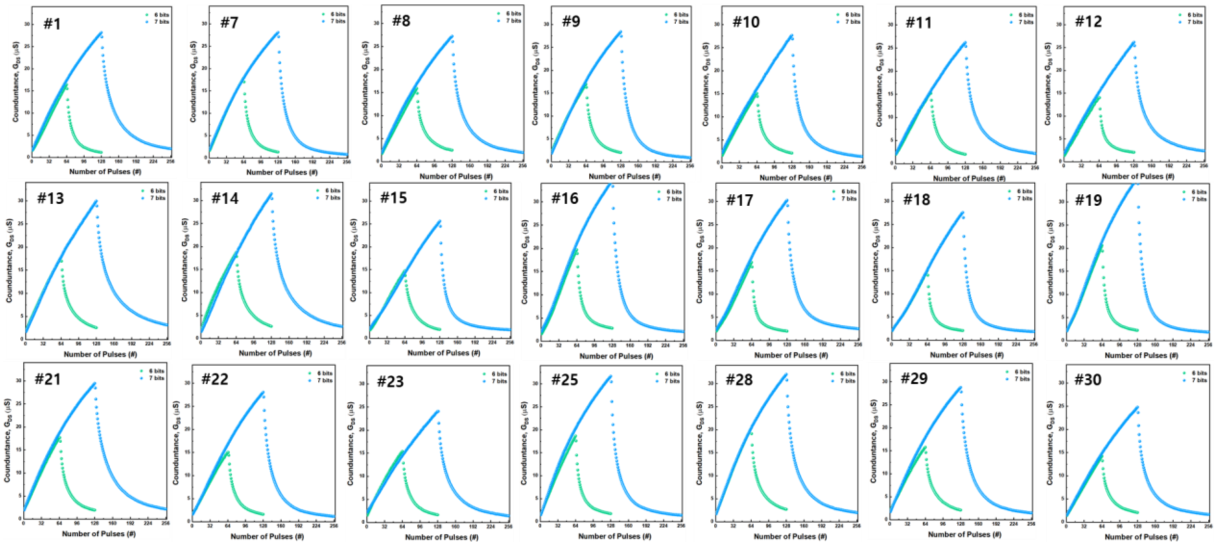

Figure S12. The characteristics of the PD curve for a 6-bit and 7-bit operation of MoS_2-EGST devices (Al Hybrid-26) with a randomly chosen 30 devices.

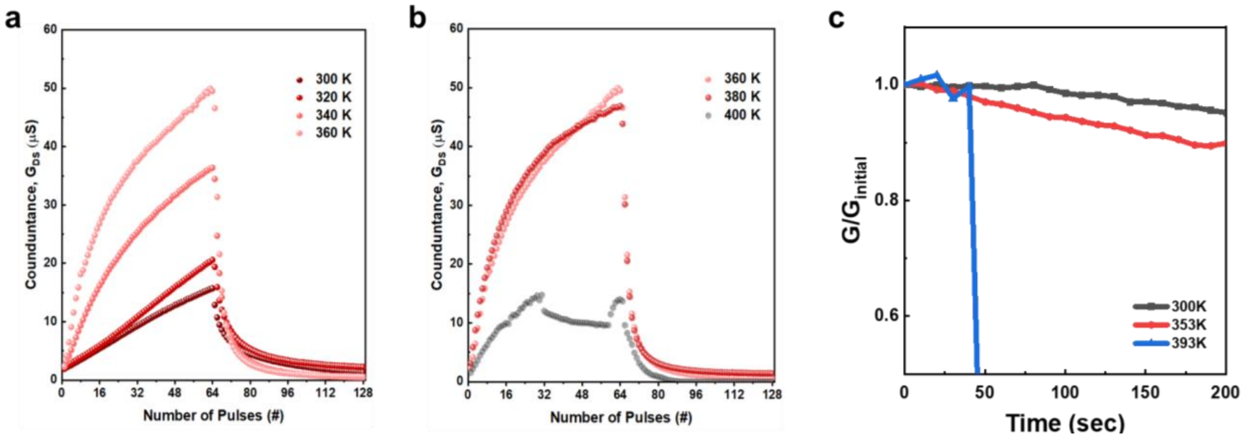

Figure S13. Variation of synaptic properties of MoS_2-EGST device at different temperature ranges (a) from 300K to 360K and (b) from 360K to 400K. (c) Retention property of MoS_2-EGST synaptic device at a temperature of 300K, 353K, and 393K.

In **Figure SX**, we analyzed the synaptic property of the MoS_2-EGST device as increasing temperature from 300K to 400K. The synaptic property is enhanced as temperature increases from 300K to 360K. This is because an increase in temperature promotes more active hopping of protons, potentially leading to an increase in the conductance ratio. But above 360K, conductance ratio starts to decrease as the temperature exceeds 360K, and electrolyte breakdown occurs at temperatures exceeding 400K as the EDL phenomenon relying on protons is vulnerable to decreased moisture and polymer breakdown when exposed to elevated temperatures.

Table S1. Comparison of synaptic performances with MoS2-ESGT and other artificial synapse candidates.

Device	On/off ratio	Nonlinearity (potentiation/depression)	Cycle-to-cycle varition	Device-to-device variation
MoS_2-EGST (This work)	15	0.64 / 7.13	low	low
Cation-based memristor[S1]	12	2.4/−4.88	high	-
PCMO memristor[S2]	7	3.6/−6.8	low	-
Vacancy-based Memristor[S3]	5	1.9/−0.6	high	-
FeFET[S4]	100	1.7/1.5	low	-

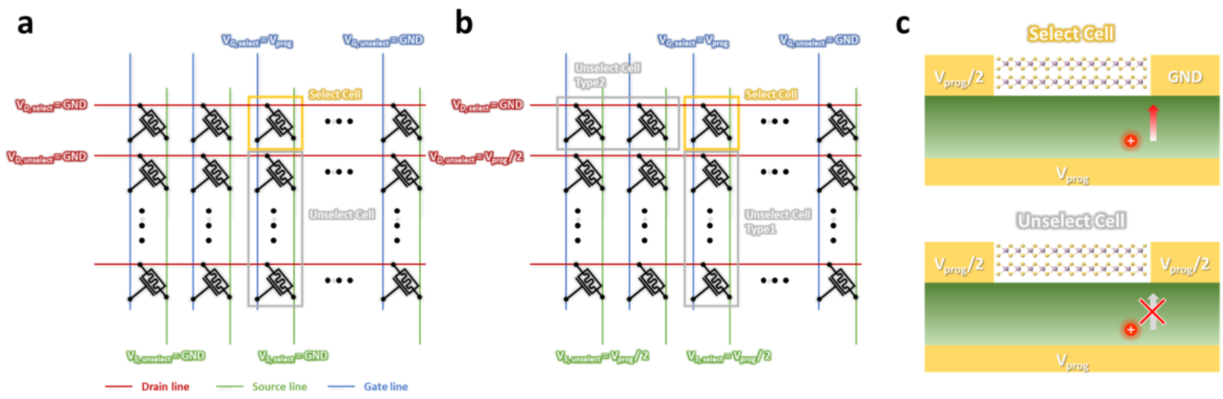

Figure S14. Programming scheme of MoS_2-EGST array. (a) Ground and (b) half-voltage programming scheme. The ground scheme cannot address a single cell due to unwanted conductance updates at unwanted cells. (c) The bias of the accessed cells in a half-voltage scheme. Conductance change occurs at the selected cell with enough V_{DS}, however, unselected cell cannot modulate conductance with lowered V_{DS} due to half-programming bias is applied to the drain.

References

[S1] S. H. Jo, T. Chang, I. Ebong, B. B. Bhadviya, P. Mazumder, W. Lu, *Nano Lett.* **2010**, 10, 1297.

[S2] S. Park, A. Sheri, J. Kim, J. Noh, J. Jang, M. Jeon, B. Lee, B. Lee, B. Lee, H.-J. Hwang, "Neuromorphic speech systems using advanced ReRAM-based synapse", presented at *2013 IEEE International Electron Devices Meeting (IEDM)*, **2013**.

[S3] M. Jerry, P.-Y. Chen, J. Zhang, P. Sharma, K. Ni, S. Yu, S. Datta, "Ferroelectric FET analog synapse for acceleration of deep neural network training", presented at *2017 IEEE International Electron Devices Meeting (IEDM)*, **2017**.

[S4] J. Woo, K. Moon, J. Song, S. Lee, M. Kwak, J. Park, H. Hwang, *IEEE Electron Dev. Lett.* **2016**, 37, 994.
